# Resilience and Time Perspective in a Clinical Sample of Adolescents

**DOI:** 10.3390/children13081028

**Published:** 2026-08-01

**Authors:** Marco Walg, Claudio Prado, Daniel El-Wahsch, Stephan Bender, Gerhard Hapfelmeier

**Affiliations:** 1 Department of Child and Adolescent Psychiatry and Psychotherapy, Sana Klinikum Remscheid, Burger Str. 211, 42859 Remscheid, Germany; claudio.prado@sana.de (C.P.); gerhard.hapfelmeier@sana.de (G.H.); 2 Department of Child and Adolescent Psychiatry, Psychosomatics, and Psychotherapy, Faculty of Medicine and University Hospital Cologne, University of Cologne, 50937 Cologne, Germany; daniel.el-wahsch@alumni.uni-koeln.de (D.E.-W.); stephan.bender@uk-koeln.de (S.B.)

**Keywords:** time perspective, resilience, adolescents, posttraumatic stress disorder, depression, anxiety, trauma exposure

## Abstract

**Background/Objectives**: Time perspective and resilience are both relevant to adolescent mental health, yet their association has rarely been examined in clinical adolescent populations. This study investigated associations between resilience and time perspective and tested their independent associations with depressive, anxiety, and PTSD symptoms beyond trauma-related variables. **Methods**: A total of 105 adolescents aged 14 to 20 years were recruited from a child and adolescent psychiatric outpatient department. Participants completed self-report measures of resilience, time perspective, trauma exposure, and PTSD, depressive, and anxiety symptoms. Discrepancy from the optimal balanced time-perspective profile was quantified using the revised Deviation from a Balanced Time Perspective coefficient (DBTP-r). Spearman correlations and linear regression models were computed using DBTP-r alone, DBTP-r plus resilience, and extended models including trauma-related variables. **Results**: Higher DBTP-r was associated with lower resilience. Higher resilience was associated with higher Future and Past Positive scores and lower Past Negative scores. DBTP-r accounted for substantial variance in symptom severity. When resilience was added, both DBTP-r and resilience were independently associated with depressive, anxiety, and PTSD symptoms. In the extended models, DBTP-r and resilience remained significant for all three outcomes. Of the trauma-related predictors, physical sexual abuse was associated with PTSD symptoms in the extended model, but this association was no longer significant after controlling for biological sex. The cumulative number of other potentially traumatic experiences was not independently associated with symptom severity in the extended models. **Conclusions**: Time perspective and resilience appear to be related but distinguishable constructs. Both were independently associated with symptom levels of depression, anxiety, and PTSD in adolescents.

## 1. Introduction

Hope and optimism are two central elements of resilience. Each contributes specifically to coping with stress and restoring stability after adversity or trauma [1]. Optimism denotes the generalized conviction that desirable outcomes are more probable than undesirable ones [2], whereas hope is anchored in goal pursuit and describes a positive motivational state built on determination and forward planning toward valued goals [3]. Both constructs have well-documented protective functions. Hope has been linked to constructive coping, better psychological adjustment, and higher well-being, and acts as a protective resilience factor in the face of potentially traumatic events such as adverse childhood experiences [4,5,6,7,8]. Optimism, in turn, has been associated with greater well-being under stress and with adaptive, flexible responses to potentially traumatic experiences [5,9,10,11].

Although hope and optimism are distinct constructs, they share a defining characteristic: a positive future orientation. This orientation is also central to time perspective, a psychological framework that structures experience across past, present, and future. Thus, positive future orientation provides a conceptual link between resilience and time perspective, although these areas have largely been studied separately. Zimbardo and Boyd described time perspective as a largely non-conscious process that sorts social and non-social experiences into separate temporal categories, the time dimensions [12]. They identified at least five dimensions: Past Negative, a pessimistic, regret-laden view of what has been; Past Positive, a warm and nostalgic stance toward the past, often tied to tradition and cherished memories; Present Fatalistic, the belief that life is governed by fate, with feelings of helplessness and resignation; Present Hedonistic, an impulsive, pleasure-oriented focus on the here and now; and Future, a goal-directed mindset that prioritizes planning and is typically linked to delayed gratification and long-term rewards. All five dimensions can be measured with the self-report Zimbardo Time Perspective Inventory (ZTPI) [12].

The individual configuration of these time dimensions constitutes a person’s time perspective profile. A balanced time perspective reflects optimal values across the dimensions: strong Past Positive and Future orientations, a moderate Present Hedonistic orientation, and weak Past Negative and Present Fatalistic orientations [13]. The degree to which a profile departs from these optimal values can be expressed by the Deviation from a Balanced Time Perspective (DBTP) coefficient [14] and its revised form, the DBTP-r [15]. An imbalanced profile has been linked to maladaptive outcomes, including poorer physical and mental health [16], whereas a balanced profile appears to be protective [17].

Developmental theory suggests that time perspective changes during the transition from childhood to adolescence. As abstract and hypothetical reasoning develop, adolescents gradually move from a predominantly present-focused orientation toward a more integrated capacity to consider the past, present, and future [18]. Consistent with this developmental account, cross-sectional comparisons suggest that future orientation is still developing during adolescence. Adolescents reported stronger present-hedonistic and weaker future orientations than adults, indicating that future-oriented thinking has not yet reached adult levels. This pattern suggests an ongoing age-related shift in which the focus on the present is gradually expanded by an increasing future orientation [19]. At the same time, adolescents are already capable of meaningful future-directed thinking, combining enjoyment of the present with trust in a better future, which supports responsible decision-making and relational engagement [20]. Moreover, temporal cognitive framing remains open to modification during mid-to-late adolescence [21]. Mid-to-late adolescence may therefore be a particularly informative period for studying how time perspective relates to resilience and psychopathology, and findings from adult samples should not be assumed to apply directly to adolescents.

Time perspective is not only theoretically relevant but also clinically meaningful. Across psychiatric disorders, characteristic alterations in time perspective emerge, suggesting that DBTP-r may represent a transdiagnostic correlate of psychopathology. For example, individuals with posttraumatic stress typically show elevated Past Negative and Present Fatalistic scores, while Past Positive and Future scores are reduced [22,23]. This fits cognitive models of posttraumatic stress, which hold that the disorder is maintained by a disturbed sense of present and future threat and by fragmented, largely negative autobiographical memory [24,25]. Findings in adolescents converge with these accounts: in a clinical sample of 14- to 19-year-olds with posttraumatic stress, Past Negative and Present Fatalistic scores were elevated and Past Positive and Future scores reduced, with only Present Hedonistic remaining close to its optimal value [26]. Characteristic deviations from a balanced time perspective have also been reported in other vulnerable groups, such as adolescent refugees [27]. Depression likewise tends to be associated with a negative view of the past, fatalistic beliefs about the present, and a weakened future orientation [16,28]. Individuals with anxiety disorders show a similar pattern of deviation from a balanced time perspective [16,29]. These patterns extend to adolescents: those with depression show higher Past Negative and Present Fatalistic orientations and lower Past Positive and Future orientations [30], and greater anxiety has been linked to more negative temporal orientations across dimensions [31]. In a community sample of adolescents, a positive view of the past was associated with lower depression, whereas a negative view was associated with higher depression [32].

Several earlier studies have connected time perspective to resilience. In line with the constructs of hope and optimism, resilience correlates positively with a Future orientation [33]. One study of at-risk youths living in economically disadvantaged areas found that future orientation mediated the relationship between connectedness to a caring, reliable adult and resilience [34]. Another study found that resilience was positively associated with high Present Hedonistic and Future orientations and negatively associated with high Past Negative and Present Fatalistic orientations. In the same study, Past Negative and Past Positive moderated the link between resilience and meaning in life, underscoring how a positive view of the past supports resources such as resilience and the preservation of meaning [35]. A pronounced Past Negative orientation has likewise been shown to correlate significantly and negatively with mindfulness, inner peace, and resilience [36]. In a sample of Chinese students aged 15 to 19 years, a balanced time perspective correlated positively with resilience [37]. Comparable links among time perspective, learned helplessness, and resilience have recently been reported in adults exposed to prolonged warfare, indicating that time perspective and resilience are intertwined across populations and stressors [38]. Although most findings on the association between resilience and time perspective stem from adult or community samples, there is evidence that these associations are also relevant during adolescence. A recent meta-analysis showed that the relationships between individual time-perspective dimensions and well-being are generally robust across age groups [39], and a survey study found some of these associations to be even stronger at younger ages [40]. Several considerations suggest that the core patterns should not only replicate but may also be more pronounced in adolescents with mental health problems. Fatalistic present orientation is highly relevant to internalizing symptoms such as depression and anxiety [16,28] and may be particularly salient in adolescence, when orientation towards the present is developmentally more pronounced than in adulthood [18]. In addition, trauma-related mechanisms—such as a poorly elaborated, past-fixated autobiographical memory and temporal disintegration—tie a Past Negative orientation directly to symptom severity and distress [24,25,41]. This pattern also appears to extend to adolescents [26,30]. By contrast, the positive association between resilience and a Present Hedonistic orientation observed in adults may be attenuated or shifted in adolescents, because a present-hedonistic orientation is developmentally normative in adolescence, declines with age, and may be adaptive at moderate but maladaptive at high levels [42,43].

Epidemiological work suggests that roughly 62% to 68% of all children and adolescents are exposed to at least one potentially traumatic event before adulthood [44,45]. Of those exposed, about 16% go on to develop persistent, clinically relevant posttraumatic stress symptoms and to meet the criteria for posttraumatic stress disorder (PTSD) [46]. Most adolescents therefore adapt well and do not show lasting PTSD symptoms after exposure to potentially traumatic events (EPTE). Across studies of exposed adolescents, higher cumulative EPTE has been linked to more pronounced PTSD symptoms [44,47]. PTSD, however, is not the only possible sequela of childhood trauma. Exposure in young people also raises the likelihood of depressive and anxiety symptoms, and higher cumulative exposure has been associated with increased symptoms across a range of psychiatric syndromes, not PTSD alone [48,49,50]. Yet the broader impact of elevated exposure to potentially traumatic events on syndromes other than PTSD has received comparatively little attention in research on children and adolescents [49]. Several factors determine whether PTSD symptoms emerge after EPTE, and one that matters beyond cumulative exposure is the type of event [51]. Survivors of interpersonal potentially traumatic events, such as physical or sexual violence, generally show higher rates of PTSD symptoms than survivors of non-interpersonal events [52]. Interpersonal potentially traumatic events—and physical sexual abuse (PSA) in particular—are therefore associated with a heightened risk of clinically relevant PTSD symptoms or the full disorder [44,53]. Beyond posttraumatic stress, comorbid conditions such as depression and elevated suicidality have also been reported in traumatized adolescents, consistent with the view that the clinical consequences of EPTE extend well beyond PTSD [54]. Clinical samples of adolescents tend to show a higher prevalence of EPTE than general-population samples [55]. As noted above, PSA in particular raises the risk of PTSD, yet other symptom domains—most notably depressive and anxiety symptoms—can equally follow trauma [48].

Despite extensive research on resilience and on time perspective, little is known about how these two constructs relate to each other in clinical adolescent populations. Previous analyses of the same clinical sample addressed different research questions: one examined associations among trauma exposure, time perspective, posttraumatic stress symptoms, and cannabis use [26], whereas another examined potentially traumatic experiences, physical sexual abuse, and psychiatric symptom burden across several symptom domains [48]. However, these studies did not examine resilience, its association with time perspective, or the independent contributions of resilience and temporal imbalance to psychopathology. The present study therefore uses this previously studied dataset to address this distinct analytic question. Beyond this descriptive aim, we used regression analyses to examine how much variance in symptoms of PTSD, depression, and anxiety each construct—resilience and temporal imbalance—accounts for when considered separately and when examined together. In a further step, trauma-related variables were included to assess whether cumulative exposure to potentially traumatic events and physical sexual abuse accounted for additional variance beyond these psychological factors. This approach allowed us to assess whether resilience and temporal imbalance represent overlapping or distinct correlates of psychopathology in clinically referred adolescents.

## 2. Materials and Methods

### 2.1. Participants and Procedure

Participants were recruited from a child and adolescent psychiatric outpatient department in Germany during their first appointment, before treatment had begun. The initial clinical consultation lasted at least one hour and was conducted by trained and experienced psychologists and physicians. It comprised a symptom assessment through a standardized clinical interview that also covered the assessment of psychopathological findings according to the manual for the assessment and documentation of psychopathology in psychiatry [56]. Because data collection for the study took place immediately after this first consultation, no confirmed diagnoses were available at that point.

All patients between 14 and 20 years of age were invited to take part. Exclusion criteria comprised psychotic symptoms, dyslexia, and below-average cognitive ability, judged on the basis of the clinical impression formed during the first consultation and a review of school reports. These criteria were applied to ensure that every participant could work through the survey independently and adequately, thereby safeguarding the highest possible data quality. Acute psychotic symptoms were evaluated as part of the standardized clinical interview and the assessment of psychopathological findings during the initial consultation. Dyslexia and clearly below-average cognitive ability were assessed on the basis of the clinical impression formed during the first consultation and a review of school reports. For the assessment of reading and spelling abilities, both current school reports and primary school reports were required.

The study followed a cross-sectional design. Informed consent was secured from all participating adolescents as well as from their legal guardians. Under the supervision of a clinic staff member, patients completed a single online survey administered with the LamaPoll software. Data collection ran from November 2023 to August 2024. The present analyses are based on the same clinical adolescent sample that was included in two previous publications: one focusing on time perspective in relation to posttraumatic stress and cannabis use [26], and another describing cumulative trauma exposure, physical sexual abuse, and psychiatric symptom burden [48]. The present study addresses a distinct research question by introducing resilience, examining its association with time perspective, and testing the independent associations of resilience and deviation from a balanced time perspective with depressive, anxiety, and PTSD symptoms in multivariable models. Recruitment began after the study had received a favorable opinion from the Research Ethics Committee of the North Rhine State Chamber of Physicians (Ärztekammer Nordrhein), Germany, in April 2023 (reference 2023048).

### 2.2. Measures

#### 2.2.1. Child and Adolescent Trauma Screen–2 (CATS-2)

The German adaptation of the Child and Adolescent Trauma Screen–2 (CATS-2) [57] is a self-report questionnaire that captures cumulative EPTE and posttraumatic stress symptoms in children and adolescents in line with the ICD-10 [58] and the DSM-5 [59]. Using a checklist of 15 distinct potentially traumatic events, among them PSA, respondents indicate on a dichotomous scale whether or not they have experienced each event. Thus, the cumulative EPTE score reflects the number of different categories of lifetime potentially traumatic events endorsed and does not capture the frequency, duration, severity, or timing of traumatic experiences. The level of posttraumatic stress symptoms is then assessed with 25 items that rate symptom frequency on a four-point Likert scale (0 = never; 3 = almost always). A total symptom score of ≥25 indicates clinically relevant PTSD symptomatology. In the present sample, the CATS-2 demonstrated high internal consistency (α = 0.94).

#### 2.2.2. Center for Epidemiologic Studies Depression Scale—Revised (CESD-R)

Depressive symptoms were measured with the German version of the Center for Epidemiologic Studies Depression Scale—Revised (CESD-R) [60], a self-report instrument that taps cognitive, affective, and behavioral features of depressive disorders. For each item, respondents indicate how frequently the described symptom occurred, on a five-point scale ranging from (0) not at all or for less than one day to (4) nearly every day over the preceding two weeks. A total score of ≥16 indicates clinically relevant depressive symptomatology. In the present sample, the CESD-R demonstrated high internal consistency (α = 0.91).

#### 2.2.3. Manifest Anxiety Subscale (MA)

General anxiety was assessed with the Manifest Anxiety (MA) subscale of the Anxiety Questionnaire for Students [61], which gauges the degree of anxiety symptoms that may surface on a physical, emotional, and cognitive level. The subscale comprises 13 statements, each answered on a dichotomous scale indicating agreement (0) or disagreement (1). A percentile rank > 88 was used as an indicator of clinically relevant anxiety. In the present sample, the MA subscale demonstrated high internal consistency (α = 0.87).

#### 2.2.4. Zimbardo Time Perspective Inventory (ZTPI) and Deviation from a Balanced Time Perspective (DBTP-r)

Time perspective was assessed with a German short form of the ZTPI [62], in which each of the five dimensions is represented by two items rated on a five-point Likert scale (1 = very untrue; 5 = very true). A mean score was computed for every subscale (PN = Past Negative, PP = Past Positive, PF = Present Fatalistic, PH = Present Hedonistic, F = Future). The DBTP-r index was derived using the formula proposed by Jankowski et al. [15] to quantify the balance of an individual’s time perspective: In the validation study, the German short form was reported to show sufficient reliability and validity for assessing all five time-perspective dimensions [62].

$$DBTP-r=\sqrt{{\left(oPN-ePN\right)}^{2}+{\left(oPP-ePP\right)}^{2}+{\left(oPF-ePF\right)}^{2}+{\left(oPH-ePH\right)}^{2}+{\left(oF-eF\right)}^{2}}$$

The optimal values are *oPN* = 1.00, *oPP* = 5.00, *oPF* = 1.00, *oPH* = 3.40, and *oF* = 5.00 [15], while the empirical scores (for example, *ePN*) correspond to the mean values of the respective scales. A DBTP-r of 0 denotes a fully balanced time perspective, and higher values reflect a greater departure from the optimal balanced profile. Internal consistencies for the two-item ZTPI subscales ranged from low to acceptable and were broadly comparable to those reported in the validation study [62]. Specifically, Spearman–Brown coefficients were r_SB_ = 0.67 for Past Negative, r_SB_ = 0.56 for Past Positive, r_SB_ = 0.45 for Present Fatalistic, r_SB_ = 0.57 for Present Hedonistic, and r_SB_ = 0.62 for Future.

#### 2.2.5. Resilience Scale–13 (RS-13)

Resilience was assessed with the Resilience Scale–13 (RS-13) [63], a self-report instrument designed to measure psychological resilience. Resilience is conceptualized as a positive personal characteristic reflecting individual adaptability. Within this framework, resilience is assumed to moderate negative affect and perceived stress and to support flexible adaptation to difficult life circumstances. The 13 items of the RS-13 assess the factors of personal competence and acceptance of self and life. Items are rated on a seven-point Likert scale ranging from 1 (strongly disagree) to 7 (strongly agree), with higher scores indicating greater resilience. In the present sample, the RS-13 demonstrated high internal consistency (α = 0.93).

### 2.3. Statistical Analyses

All analyses were conducted using the Statistical Package for the Social Sciences (SPSS, version 29.0.2.0) for Windows. Global sum scores were computed for all psychopathological syndrome scales. Physical sexual abuse (PSA) was coded dichotomously (experienced vs. not experienced) based on the corresponding item of the CATS-2 event list, and the cumulative number of other potentially traumatic events (EPTE) was calculated as the sum of all endorsed events excluding PSA. An a priori power analysis was conducted using G*Power 3.1 for the largest linear multiple regression model, including four predictors. Assuming a medium effect size (*f*^2^ = 0.15), an α-level of 0.05, and a desired statistical power of 1 − *β* = 0.85, the required sample size was *N* = 95. The final sample comprised 105 participants, and the regression analyses were based on 104 complete cases, thereby exceeding the a priori target.

Prior to analysis, the distribution of all variables was examined using the Shapiro–Wilk test and visual inspection of Q–Q plots. Because resilience scores deviated significantly from a normal distribution (*p* < 0.05), the association between resilience and DBTP-r was evaluated using Spearman rank-order correlations.

To examine the associations of temporal imbalance and resilience with clinical symptoms, we computed three sets of regression models for each outcome variable (manifest anxiety, PTSD symptoms, and depression), resulting in nine models in total.

**Model Set 1 (single-predictor models)**: For each outcome, DBTP-r was entered as the sole statistical predictor to quantify the bivariate association between temporal imbalance and symptom severity.

**Model Set 2 (two-predictor models)**: For each outcome, DBTP-r and resilience were entered simultaneously to assess their shared and unique associations with symptom severity.

**Model Set 3 (extended models including trauma-related variables)**: For each outcome, DBTP-r, resilience, PSA, and the cumulative number of other EPTE were entered to evaluate whether trauma exposure accounted for additional variance beyond the psychological variables.

This analytic strategy allowed us to determine (a) the variance in symptom severity accounted for by temporal imbalance alone, (b) the joint and unique associations of temporal imbalance and resilience with symptom severity, and (c) the incremental variance accounted for by interpersonal and cumulative trauma exposure. Due to one missing value in the variables included in the regression analyses, the regression models were based on 104 complete cases. In line with the cross-sectional design, the terms predictor and prediction are used throughout in a purely statistical sense, referring to variables entered into the regression models, and do not imply causality or temporal precedence.

Assumptions of linearity, homoscedasticity, and approximate normality of residuals were evaluated for all nine regression models using standard diagnostic plots. Potentially influential observations were assessed via Cook’s distance and leverage values. The diagnostic checks indicated no material departures from linearity, homoscedasticity, or residual normality; all Cook’s distance values were below 0.15 and all leverage values were below 0.20, suggesting the absence of influential observations.

## 3. Results

### 3.1. Sample Characteristics

A total of 105 patients participated in the study. On average, they were approximately 16 years of age (*M* = 15.5; *SD* = 1.3) and were predominantly biologically female (73%); the remaining participants were male (25%) or diverse (2%).

As shown in Table 1, participants reported exposure to a broad spectrum of potentially traumatic events. The most frequently endorsed events were bullying (54.8%), experiencing violence in the community (48.1%), observing violence in the community (41.3%), and serious accidents (41.3%). Physical sexual abuse was reported by 34.6% of the sample. On average, participants had experienced 4.4 (*SD* = 2.9) potentially traumatic event categories, and 4.0 (*SD* = 2.7) when physical sexual abuse was excluded.

#### Clinically Relevant Symptom Levels and Preliminary Suspected ICD-10 Diagnoses

Based on the cut-off values described above for the respective self-report instruments, 97 patients (92%) showed clinically relevant depressive symptoms, 66 patients (63%) reported clinically relevant PTSD symptoms, and 54 patients (51%) showed clinically relevant general anxiety symptoms.

After the first appointment at the child and adolescent psychiatric outpatient department and completion of the study assessment, preliminary suspected ICD-10 diagnoses were assigned by experienced clinicians. More than one preliminary suspected primary diagnosis could be assigned per patient. The suspected ICD-10 diagnoses assigned most frequently were as follows: 49 patients (46.7%) received a suspected diagnosis of a depressive episode (F32) of varying severity, ranging from mild to severe without psychotic symptoms (F32.0, F32.1, F32.2); 30 patients (28.6%) received a suspected diagnosis of attention-deficit/hyperactivity disorder (F90.0); 17 patients (16.2%) received a suspected diagnosis of social phobia (F40.1); 12 patients (11.4%) received a suspected diagnosis of PTSD (F43.1); and 7 patients (6.7%) received a suspected diagnosis of borderline personality disorder (F60.31). Due to restrictions imposed by the responsible ethics committee, final confirmed diagnoses could not be accessed for the present study.

### 3.2. Relationship Between Resilience and Time Perspective

The RS-13 total score correlated significantly and negatively with DBTP-r, which indicates that a greater deviation from a balanced time perspective was accompanied by lower resilience (*r*_s_ = −0.44, *p* < 0.001).

As reported in Table 2, the observed temporal profile departed from the optimal values on several dimensions: participants scored higher on Past Negative and Present Fatalistic and lower on Past Positive and Future, whereas Present Hedonistic lay comparatively close to its optimal value.

Table 2 further shows that the RS-13 total score correlated significantly and positively with Past Positive (*r*_s_ = 0.22, *p* = 0.02) and Future (*r*_s_ = 0.35, *p* < 0.001) and significantly and negatively with Past Negative (*r*_s_ = −0.20, *p* = 0.04), while no significant associations emerged for Present Fatalistic (*r*_s_ = −0.19, *p* = 0.05) or Present Hedonistic (*r*_s_ = 0.02, *p* = 0.85). Higher resilience was thus accompanied by stronger Past Positive and Future orientations and a weaker Past Negative orientation.

### 3.3. Regression Analyses of Psychopathological Symptoms

#### 3.3.1. Single-Predictor Models (DBTP-r Only)

Regression analyses with DBTP-r as the sole statistical predictor showed that temporal imbalance was significantly associated with all three clinical outcomes. As shown in Table 3, DBTP-r was associated with depressive symptoms (*β* = 0.60, *p* < 0.001), anxiety symptoms (*β* = 0.50, *p* < 0.001), and PTSD symptoms (*β* = 0.60, *p* < 0.001). These models accounted for 33% of the variance in depression (adjusted R^2^ = 0.33), 29% in anxiety (adjusted R^2^ = 0.29), and 38% in PTSD symptoms (adjusted R^2^ = 0.38).

#### 3.3.2. Two-Predictor Models (DBTP-r and Resilience)

When DBTP-r and resilience were entered simultaneously, both constructs were significantly and independently associated with all three outcomes (Table 4). Greater temporal imbalance and lower resilience were associated with more severe depressive symptoms (*β* = 0.40 and *β* = −0.40, both *p* < 0.001), anxiety symptoms (*β* = 0.40 and *β* = −0.30, both *p* < 0.01), and PTSD symptoms (*β* = 0.50 and *β* = −0.30, both *p* < 0.01). These models accounted for 43% of the variance in depression (adjusted R^2^ = 0.43), 37% in anxiety (adjusted R^2^ = 0.37), and 44% in PTSD symptoms (adjusted R^2^ = 0.44).

Because resilience and DBTP-r were significantly correlated (*r* = −0.44, *p* < 0.001), collinearity diagnostics were conducted for the predictor set used in the regression analyses. Across all models, the tolerance values were 0.81 and the VIF values were 1.2. These indices are well within recommended thresholds (tolerance > 0.10; VIF < 10). These values did not indicate problematic multicollinearity.

#### 3.3.3. Extended Models Including Trauma-Related Variables

As presented in Table 5, the full models including DBTP-r, resilience, PSA, and EPTE were significant for all outcomes. For depressive symptoms, DBTP-r (*β* = 0.40, *p* < 0.001) and resilience (*β* = −0.40, *p* < 0.001) remained significant statistical predictors, whereas PSA and EPTE showed no significant associations. The model accounted for 42% of the variance (adjusted R^2^ = 0.42).

For anxiety symptoms, DBTP-r (*β* = 0.30, *p* < 0.01) and resilience (*β* = −0.30, *p* < 0.01) again emerged as significant statistical predictors, while PSA and EPTE did not. The model accounted for 35% of the variance (adjusted R^2^ = 0.35).

For PTSD symptoms, DBTP-r (*β* = 0.40, *p* < 0.001), resilience (*β* = −0.30, *p* < 0.01), and PSA (*β* = 0.20, *p* < 0.01) were significant statistical predictors, whereas EPTE was not. This model accounted for 50% of the variance (adjusted R^2^ = 0.50).

Compared to the two-predictor models, the extended model accounted for more variance in PTSD symptoms (from adjusted R^2^ = 0.44 to 0.50), indicating that PSA accounted for additional variance beyond temporal imbalance and resilience specifically in PTSD symptoms. For depressive and anxiety symptoms, the adjusted R^2^ values did not increase after trauma-related variables were added. Taken together, the hierarchical model structure showed that temporal imbalance and resilience accounted for the largest share of the variance across outcomes, whereas trauma-related variables—particularly PSA—accounted for incremental variance primarily in PTSD symptoms.

To address the potential influence of demographic variables, we conducted additional sensitivity analyses in which age and biological sex were added as covariates to the extended regression models. Because biological sex was entered as a dichotomous variable (female = 0, male = 1), the two participants categorized as diverse were excluded from these analyses. In the sensitivity analysis for PTSD symptoms, DBTP-r remained a significant positive predictor (*β* = 0.38, *p* < 0.001), and resilience remained a significant negative predictor (*β* = −0.23, *p* = 0.006). Biological sex also emerged as a significant predictor (*β* = −0.21, *p* = 0.010), indicating lower PTSD symptom scores among male compared with female participants. Age was not significant (*β* = 0.09, *p* = 0.188). After age and biological sex were added, PSA no longer reached statistical significance (*β* = 0.13, *p* = 0.108), and other EPTE without PSA remained non-significant (*β* = 0.12, *p* = 0.170). The PTSD sensitivity model was significant, *F*(6, 95) = 20.00, *p* < 0.001, and explained 53% of the variance in PTSD symptoms (adjusted R^2^ = 0.53).

In the corresponding sensitivity analysis for depressive symptoms, DBTP-r remained a significant positive predictor (*β* = 0.37, *p* < 0.001), and resilience remained a significant negative predictor (*β* = −0.31, *p* < 0.001). Biological sex also emerged as a significant predictor (*β* = −0.27, *p* = 0.002), indicating lower depressive symptom scores among male compared with female participants. Age (*β* = 0.10, *p* = 0.190), PSA (*β* = −0.08, *p* = 0.367), and other EPTE without PSA (*β* = 0.03, *p* = 0.701) were not significant. The depression sensitivity model was significant, *F*(6, 95) = 16.67, *p* < 0.001, and explained 48% of the variance in depressive symptoms (adjusted R^2^ = 0.48).

In the corresponding sensitivity analysis for anxiety symptoms, DBTP-r remained a significant positive predictor (*β* = 0.29, *p* = 0.004), and resilience remained a significant negative predictor (*β* = −0.22, *p* = 0.017). Biological sex also emerged as a significant predictor (*β* = −0.29, *p* = 0.002), indicating lower anxiety symptom scores among male compared with female participants. Age (*β* = −0.03, *p* = 0.673) and PSA (*β* = −0.01, *p* = 0.919) were not significant. Other EPTEs without PSA showed a positive but non-significant association with anxiety symptoms (*β* = 0.16, *p* = 0.086). The anxiety sensitivity model was significant, *F*(6, 95) = 12.28, *p* < 0.001, and explained 40% of the variance in anxiety symptoms (adjusted R^2^ = 0.40).

Overall, these sensitivity analyses indicate that the main findings for DBTP-r and resilience remained robust after controlling for age and biological sex, although biological sex showed additional associations with symptom severity and the association between PSA and PTSD symptoms no longer reached statistical significance.

## 4. Discussion

The present results align with earlier work showing that a more balanced time perspective is associated with higher resilience [37]. In line with this literature, a greater deviation from the optimal time perspective (a higher DBTP-r) was related to lower resilience in this clinical sample of adolescents. Notably, the correlation found here (*r* = −0.44) was stronger than the small effect reported in an adolescent sample aged 15 to 19 years (*r* ≈ 0.15). The stronger association observed here may reflect the clinical nature of the sample in two complementary ways. At the psychometric level, previous studies suggest that clinical samples may be characterized by more extreme scores and greater variability across maladaptive time-perspective dimensions, including deviation from a balanced time perspective [16,29]. Such greater variability may have reduced range restriction, thereby partly accounting for the stronger correlation with resilience observed here. At the psychopathological level, the stronger association may reflect an especially close link between future- and past-oriented dimensions, clinical symptomatology, and resilience. Across posttraumatic stress, depression, and anxiety, symptom severity has been associated particularly with elevated Past Negative and reduced Future orientation, often alongside higher Present Fatalistic and lower Past Positive orientation [16,22,23,24,25,28,29,30,31]. Taken together, this prior evidence suggests that the stronger correlation observed here may reflect the overlap between symptom-relevant temporal orientations and core aspects of resilience, particularly the ability to move beyond negative past experiences and maintain a future-oriented outlook.

A closer look at the associations between resilience and the individual ZTPI dimensions further supports the relevance of a future-oriented stance. Future orientation stood out most clearly as a correlate of resilience: higher resilience was tied to a stronger focus on what lies ahead. This finding is in line with theoretical conceptualizations of resilience that incorporate optimism, goal-directedness, and the capacity to maintain confidence in a better future [64]. The data also indicate that the appraisal of past experiences bears on resilience. Resilience correlated negatively with Past Negative and positively with Past Positive, which suggests that more resilient adolescents are less inclined to view the past in mainly aversive or regret-laden terms and are better able to draw on positive, supportive memories. This pattern is consistent with work showing that a pronounced Past Negative orientation correlates significantly and negatively with resilience [36]. Lasota and Mróz [35] did not find a direct association between Past Positive and resilience, but their results still highlight the importance of a positive past orientation: Past Positive amplified the effect of resilience on meaning in life, whereas Past Negative had the opposite effect, limiting how far resilience translated into a sense of meaning. In their model, a negative view of the past both lowered meaning in life directly and constrained the functional expression of resilience. By contrast, resilience showed no significant association with either present-oriented dimension in the current clinical sample—a pattern that differs from results in psychologically healthy adults, among whom resilience related positively to Present Hedonistic and negatively to Present Fatalistic orientation [35]. One interpretation is that, in adolescents receiving psychiatric care, resilience may be more closely bound to future-oriented cognition and to the appraisal of the past than to present-oriented dimensions. Beyond this, the present-oriented dimensions may carry a different functional meaning in adolescents than in adults. Present Hedonistic and Present Fatalistic orientations are shaped by the protracted maturation of the adolescent brain, in which an early-maturing reward-driven system and a still-developing prefrontal control system produce heightened reward seeking and normative impulsivity that decline only into adulthood [65,66]. A present-hedonistic orientation is correspondingly more pronounced in adolescence and decreases with age [42]. Moderate present-hedonism may therefore reflect age-related variation in adolescents rather than serving as a clear marker of resilient functioning. Under these conditions, present-oriented dimensions may be less differentiating with respect to resilience in adolescents than in adults, which could account for the absence of the adult pattern in the present clinical sample.

A conceptually important question is whether a balanced time perspective, and future orientation in particular, should be treated as a component of resilience or as a related but separate construct. The present findings allow for two complementary interpretations. On the one hand, future orientation overlaps considerably with attributes widely regarded as central to resilience, including optimism, hope, goal-directedness, planning, and the ability to keep a constructive outlook under stress or uncertainty [1,8,64]. From this angle, a stronger future orientation can be seen as one facet of resilient functioning. The observed correlation between resilience and future orientation supports this interpretation and suggests that resilient functioning may rest in part on a time perspective that helps individuals remain oriented toward future possibilities. On the other hand, a balanced time perspective appears to reach beyond resilience. Resilience is generally defined in terms of adaptation, recovery, or the maintenance of functioning under adversity [64], whereas a balanced time perspective refers more specifically to the temporal organization of experience across past, present, and future. It includes not only future-directedness but also the evaluative representation of the past and the capacity to switch flexibly between temporal frames as the situation requires [13]. This broader temporal architecture is not fully captured by resilience scales such as the RS-13. The present findings therefore support the view that the two constructs overlap without being identical: although DBTP-r was significantly associated with resilience, both variables were independently associated with depressive, anxiety, and PTSD symptoms. The independent associations of DBTP-r and resilience in the regression models argue against treating balanced time perspective as merely a subcomponent of resilience. A balanced time perspective may instead represent an additional, clinically meaningful framework for understanding how adolescents process adversity, organize autobiographical experience, and stay oriented toward future goals amid psychological distress.

The regression analyses showed a similar pattern across depression, anxiety, and PTSD symptoms. DBTP-r was associated with all three outcomes when entered alone. When resilience was added, both variables remained independently associated with symptom severity, and the models accounted for more variance. This pattern persisted after trauma-related variables were included. Thus, temporal imbalance and resilience appear to represent related but distinct correlates of clinical symptom burden: DBTP-r reflects the temporal organization of experience, whereas resilience reflects perceived adaptive capacity under stress.

The trauma-related variables, by contrast, showed a more limited and outcome-specific pattern in the extended models. Physical sexual abuse (PSA) accounted for incremental variance only in PTSD symptoms, whereas it showed no unique association with depressive or anxiety symptoms after DBTP-r, resilience, and other EPTE were controlled. This finding is consistent with evidence that interpersonal and sexual forms of trauma are particularly relevant for posttraumatic stress reactions [51,52,53,67]. However, the association between PSA and PTSD symptoms no longer reached statistical significance in sensitivity analyses controlling for age and biological sex. These findings should not be taken to suggest that PSA is clinically less relevant; instead, they indicate that, in the present cross-sectional models, DBTP-r and resilience explained symptom severity more consistently than the available checklist-based PSA indicator. PSA was coded dichotomously from the CATS-2 event list, which records whether such an event was reported but does not assess clinical parameters such as frequency, duration, age of onset, or the relationship to the perpetrator; these parameters were therefore not available in the present data set. This coarse operationalization may have attenuated the associations of PSA with psychopathology.

In the current sample, adolescents reported on average more than four potentially traumatic event categories, which refer to lifetime exposure as assessed by CATS-2. Although this level of exposure is high, it is consistent with evidence that clinical samples of adolescents tend to show higher rates of potentially traumatic experiences than general-population samples [55]. The lack of an independent association for cumulative EPTE should be interpreted cautiously. It diverges from studies linking cumulative trauma exposure and polyvictimization to PTSD symptoms, internalizing symptoms, and broader psychopathology in adolescents [44,47,48,49,50,68,69,70]. Several factors may account for this difference. Earlier studies did not necessarily include time perspective and resilience in the same models, whereas both constructs were consistently associated with symptom severity here. In addition, the cumulative EPTE variable was based on a simple event count and therefore could not capture clinically important characteristics of trauma exposure, such as chronicity, developmental timing, interpersonal proximity, perceived threat, or subjective meaning [51,52,53]. The present analyses also examined symptom severity rather than confirmed psychiatric diagnoses, which further limits direct comparability with diagnostically based studies. Prior work indicates that only a minority of trauma-exposed adolescents develop persistent PTSD symptoms and that posttraumatic psychopathology depends on several factors beyond the number and type of traumatic events [46]. In this context, resilience and DBTP-r may help characterize individual differences in symptom burden after trauma exposure. Future studies should use longitudinal designs, diagnostically confirmed outcomes, and more differentiated trauma assessments to examine how cumulative exposure, trauma characteristics, resilience, and time perspective jointly relate to adolescent psychopathology.

Taken together, the findings suggest that DBTP-r and resilience capture broad psychological correlates of symptom severity. The current results may also contribute to understanding why many adolescents do not develop clinically relevant symptoms after trauma exposure [46], by suggesting that resilience and time perspective may be relevant to individual differences in symptom levels after such exposure. This view is consistent with previous work showing that time perspective statistically mediated associations of trauma exposure with PTSD symptoms [26] and of early life trauma with depressive symptom severity [71]. Future longitudinal studies should therefore examine resilience and time perspective in trauma-exposed youth to clarify whether these constructs help explain the development or absence of mental health problems over time.

This study has several limitations. The cross-sectional design precludes causal conclusions about the direction of the observed associations. It remains open whether greater deviation from a balanced time perspective and lower resilience contribute to psychopathological symptoms, whether symptom severity alters temporal orientation and self-perceived resilience, or whether these relationships are reciprocal. In addition, third variables that were not assessed in the present study, such as overall illness severity or executive functioning, may influence both time perspective and resilience as well as symptom severity and may therefore account for part of the observed associations. Accordingly, the regression results should be interpreted as cross-sectional statistical associations rather than as evidence of directional effects. Moreover, several potentially relevant demographic and clinical covariates were not available for the present analyses, including socioeconomic status, time elapsed since traumatic events, final confirmed diagnoses, and detailed treatment or medication history. The sample was a convenience sample recruited exclusively from a single child and adolescent psychiatric outpatient center, which may limit the generalizability of the results to other clinical settings and to non-clinical populations. Furthermore, although the sample size of 105 participants is adequate for the regression models reported here, which included up to four statistical predictors, it is not sufficient for more complex analyses or for subgroup analyses, for example, separate models by gender, age group, or type of trauma exposure. The sample was predominantly biologically female. The predominance of biologically female participants is consistent with recent German health care data showing that, from approximately age 15 onward, female adolescents are markedly overrepresented among those receiving psychotherapeutic or psychiatric care, a pattern likely related to the increase in internalizing disorders during mid- to late adolescence and their higher epidemiological prevalence among girls [72]. Although the main findings for DBTP-r and resilience remained robust in sensitivity analyses controlling for biological sex, this variable also emerged as an additional significant predictor of symptom severity, with lower symptom scores among male compared with female participants. Given the gender imbalance in the sample, however, sufficiently powered moderation analyses could not be conducted. Future studies with larger and more balanced samples should examine whether gender moderates the associations between time perspective, resilience, and internalizing symptoms. Because no confirmed clinical diagnoses were available at the time of assessment, the analyses refer to symptom severity rather than diagnostically established disorders. The present analyses are based on the same clinical adolescent sample that was used in earlier publications on time perspective, trauma exposure, and psychopathology [26,48]. Although the present research question is distinct and introduces resilience as a new construct, this sample overlap should be considered when interpreting the contribution of the study. Time perspective was assessed with a short version of the ZTPI containing only two items per dimension. Although this reduced participant burden in a clinical sample, it may have limited the reliability, construct coverage, and validity of the individual time-perspective dimensions and, by extension, the precision of the DBTP-r estimate. Because DBTP-r is an aggregated profile-based index, the present analyses also do not disentangle the relative contribution of individual ZTPI dimensions to symptom severity. Given the brevity of the ZTPI subscales and the sample size, dimension-level regression models were not pursued because they would have increased the risk of unstable estimates and overinterpretation. Future studies should therefore replicate the present findings with the full-length ZTPI or other comprehensive measures of time perspective, ideally in larger clinical samples that allow dimension-level analyses. Moreover, all key variables were assessed by self-report, which raises the possibility of shared method variance, recall bias, and symptom-congruent response tendencies. Finally, trauma exposure beyond physical sexual abuse was operationalized as the cumulative number of potentially traumatic events, a comparatively coarse index that does not capture severity, chronicity, developmental timing, interpersonal proximity, or subjective meaning. Likewise, physical sexual abuse was coded dichotomously from the CATS-2 event list, capturing whether such an event was reported but not its frequency, duration, context, or perceived severity. Future research should therefore use longitudinal designs, include diagnostically confirmed clinical data, and apply more differentiated assessments of trauma exposure. Future studies should also examine whether changes in time perspective and resilience over the course of treatment predict changes in depressive, anxiety, and PTSD symptoms.

## 5. Conclusions

The present findings highlight the potential importance of time perspective as a clinically relevant framework for understanding adolescent psychopathology. In this sample of adolescents receiving psychiatric care, a greater deviation from a balanced time perspective was associated with lower resilience and with more severe depressive, anxiety, and PTSD symptoms. At the same time, the results indicate that a balanced time perspective is not merely a facet of resilience; the two constructs appear to share common elements, particularly in the realm of future-oriented cognition, while remaining empirically distinguishable in their associations with symptom severity. Within the present cross-sectional regression models, DBTP-r and resilience accounted for symptom variance more consistently than the available checklist-based trauma indicators. This should not be interpreted as evidence that trauma exposure is clinically less relevant. Rather, it suggests that resilience and time perspective may be relevant factors in understanding the emergence and severity of psychopathological symptoms, while also underscoring the need for more differentiated assessments of trauma characteristics to clarify how cumulative exposure, trauma type, and psychological factors jointly relate to adolescent psychopathology. Clinically, the results point to the value of assessing not only trauma history and symptom burden but also adolescents’ time perspective profile and perceived resilience. The present findings suggest that time perspective may provide an additional clinically meaningful framework for understanding adolescents’ responses to adversity and may warrant greater consideration in psychotherapy.

## Figures and Tables

**Table 1:** Frequency of exposure to potentially traumatic event categories in the sample (*N* = 105).

Potentially Traumatic Event Category	Frequency, *n* (%)
Bullying	57 (54.8)
Experiencing violence in the community	50 (48.1)
Observing violence in the community	43 (41.3)
Serious accident	43 (41.3)
Physical sexual abuse	36 (34.6)
Experiencing violence at home	35 (33.7)
Sudden death of a close person	32 (30.8)
Online sexual abuse	32 (30.8)
Observing violence at home	31 (29.8)
Other stressful event	24 (23.1)
Cyberbullying	23 (22.1)
Serious natural disaster	16 (15.4)
Scary medical procedures	15 (14.4)
Attack or physical injury	14 (13.5)
Being in a war zone	2 (1.9)
	*M (SD)*
Average number of potentially traumatic event categories	4.4 (2.9)
Average number of potentially traumatic event categories without physical sexual abuse	4.0 (2.7)

*Note.* Values for individual event categories are frequencies, *n* (%); values for the average number of event categories are *M (SD)*. The event counts refer to the number of different categories of lifetime potentially traumatic events endorsed on the Child and Adolescent Trauma Screen–2 (CATS-2) checklist and do not indicate the frequency, duration, or severity of traumatic experiences. Multiple responses were possible; percentages therefore do not sum to 100.

**Table 2:** Optimal and observed values on the dimensions of the Zimbardo Time Perspective Inventory and their correlations with resilience.

Time Dimension	Optimal Value	Observed Value, *M (SD)*	*r*_s_	*p*
Past Negative	1.00	4.1 (0.8)	−0.20	0.04
Past Positive	5.00	2.5 (0.8)	0.22	0.02
Present Fatalistic	1.00	2.9 (0.8)	−0.19	0.05
Present Hedonistic	3.40	3.3 (0.9)	0.02	0.85
Future	5.00	2.5 (0.8)	0.35	<0.001

*Note.* Optimal values follow the revised deviation from a balanced time perspective (DBTP-r) [15]. Correlations (*r*_s_) are Spearman rank-order correlations between each time dimension and resilience as measured by the RS-13 total score.

**Table 3:** Single-predictor regression analyses examining associations between deviation from a balanced time perspective and depressive, anxiety, and PTSD symptoms.

	Depression		Anxiety		PTSD Symptoms	
Statistical Predictor	*B* ± *SE*	*β*	*B* ± *SE*	*β*	*B* ± *SE*	*β*
Deviation from a balanced time perspective (*DBTP-r*)	8.4 ± 1.2	0.60 ***	14.7 ± 2.2	0.50 ***	8.0 ± 1.0	0.60 ***
Adjusted R^2^	0.33		0.29		0.38	
Model *F*	*F*(1, 102) = 51.2 ***		*F*(1, 102) = 43.6 ***		*F*(1, 102) = 63.4 ***	

*Note. B* = unstandardized regression coefficient. *SE* = standard error. *β* = standardized regression coefficient. *DBTP-r* = revised deviation from a balanced time perspective coefficient. *** *p* < 0.001.

**Table 4:** Two-predictor regression analyses examining associations of deviation from a balanced time perspective and resilience with depressive, anxiety, and PTSD symptoms.

	Depression		Anxiety		PTSD Symptoms	
Statistical Predictor	*B* ± *SE*	*β*	*B* ± *SE*	*β*	*B* ± *SE*	*β*
Deviation from a balanced time perspective (*DBTP-r*)	6.1 ± 1.2	0.40 ***	10.8 ± 2.3	0.40 **	6.4 ± 1.1	0.50 ***
Resilience	−0.3 ± 0.1	−0.40 ***	−0.5 ± 0.1	−0.30 **	−0.2 ± 0.1	−0.30 **
Adjusted R^2^	0.43		0.37		0.44	
Model *F*	*F*(2, 101) = 39.9 ***		*F*(2, 101) = 31.7 ***		*F*(2, 101) = 41.1 ***	

*Note. B* = unstandardized regression coefficient. *SE* = standard error. *β* = standardized regression coefficient. *DBTP-r* = revised deviation from a balanced time perspective coefficient. ** *p* < 0.01; *** *p* < 0.001.

**Table 5:** Extended regression analyses examining associations of deviation from a balanced time perspective, resilience, physical sexual abuse, and the cumulative number of other potentially traumatic event categories with depressive, anxiety, and PTSD symptoms.

	Depression		Anxiety		PTSD Symptoms	
Statistical Predictor	*B* ± *SE*	*β*	*B* ± *SE*	*β*	*B* ± *SE*	*β*
Deviation from a balanced time perspective (*DBTP-r*)	6.0 ± 1.3	0.40 ***	1.1 ± 0.4	0.30 **	5.5 ± 1.1	0.40 ***
Resilience	−0.3 ± 0.1	−0.40 ***	−0.7 ± 0.2	−0.30 **	−0.2 ± 0.1	−0.30 **
PSA	1.3 ± 2.4	0.04	0.6 ± 0.6	0.10	5.5 ± 2.0	0.20 **
Other *EPTE* excluding PSA	−0.2 ± 0.5	−0.01	0.2 ± 0.1	0.10	0.4 ± 0.4	0.10
Adjusted R^2^	0.42		0.35		0.50	
Model *F*	*F*(4, 99) = 19.7 ***		*F*(4, 99) = 14.7 ***		*F*(4, 99) = 26.6 ***	

*Note. B* = unstandardized regression coefficient. *SE* = standard error. *β* = standardized regression coefficient. *DBTP-r* = revised deviation from a balanced time perspective coefficient. *EPTE* = exposure to potentially traumatic events; *PSA* = physical sexual abuse. ** *p* < 0.01; *** *p* < 0.001.

## Data Availability

The data presented in this study are available on request from the corresponding author. The data are not publicly available due to privacy and ethical restrictions.
